# Differences in Blood Flow Patterns and Endothelial Shear Stress at the Carotid Artery Using Different Exercise Modalities and Intensities

**DOI:** 10.3389/fphys.2022.857816

**Published:** 2022-05-10

**Authors:** Samuel Montalvo, Manuel Gomez, Alondra Lozano, Sabrina Arias, Lisa Rodriguez, Francisco Morales-Acuna, Alvaro N. Gurovich

**Affiliations:** ^1^ Clinical Applied Physiology Lab, College of Health Sciences, The University of Texas at El Paso, El Paso, TX, United States; ^2^ Doctor of Physical Therapy Program, Rehabilitation Sciences Department, College of Health Sciences, The University of Texas at El Paso, El Paso, TX, United States

**Keywords:** atherosclerosis, blood lactate levels, aerobic exercise, resistance exercise, stroke

## Abstract

Endothelial dysfunction is the first pathophysiological step of atherosclerosis, which is responsible for 90% of strokes. Exercise programs aim to reduce the risk of developing stroke; however, the majority of the beneficial factors of exercise are still unknown. Endothelial shear stress (ESS) is associated with endothelial homeostasis. Unfortunately, ESS has not been characterized during different exercise modalities and intensities in the carotid artery. Therefore, the purpose of this study was to determine exercise-induced blood flow patterns in the carotid artery. Fourteen apparently healthy young adults (males = 7, females = 7) were recruited for this repeated measures study design. Participants completed maximal oxygen consumption (VO2max) tests on a Treadmill, Cycle-ergometer, and Arm-ergometer, and 1-repetition maximum (1RM) tests of the Squat, Bench Press (Bench), and Biceps Curl (Biceps) on separate days. Thereafter, participants performed each exercise at 3 different exercise intensities (low, moderate, high) while a real-time ultrasound image and blood flow of the carotid artery was obtained. Blood flow patterns were assessed by estimating ESS *via* Womersley’s estimation and turbulence *via* Reynold’s number (Re). Data were analyzed using a linear mixed-effects model. Pairwise comparisons with Holm-Bonferroni correction were conducted with Hedge’s g effect size to determine the magnitude of the difference. There was a main effect of intensity, exercise modality, and intensity * exercise modality interaction on both ESS (*p* < 0.001). Treadmill at a high intensity yielded the greatest ESS when compared to the other exercise modalities and intensities, while Bench Press and Biceps curls yielded the least ESS. All exercise intensities across all modalities resulted in turbulent blood flow. Clinicians must take into consideration how different exercise modalities and intensities affect ESS and Re of the carotid artery.

## Introduction

Cardiovascular (CV) diseases, including coronary artery disease and stroke, are the leading cause of death worldwide. One in every 19 deaths are produced by a stroke, and there are more than 610,000 new cases of stroke per year. The total direct and indirect costs of CV for the USA are estimated to be around 43.6 billion dollars (Urbich et al., 2020). Atherosclerosis is responsible for 9 in every 10 cases of Stroke (Qaja and Bhimji, 2017); in addition, CV comorbidities are common features among stroke survivors (Tang et al., 2009).

Endothelial dysfunction is recognized as the first step for the development of 90% of all CV diseases (Benjamin et al., 2017), is a pathological condition characterized by an unbalance between vasodilatory and vasoconstrictory mechanisms (Flammer et al., 2012), and is generally defined as the decrease in nitric oxide (NO) bio-availability within the endothelium (Harris et al., 2010). The primary physiological mechanism that regulates endothelial function in endothelial shear stress (ESS), which is the frictional force produced between blood flow and endothelial cells (Sriram et al., 2016); where increments of ESS (e.g., during exercise) are known to improve endothelial nitric oxide synthase (eNOS) gene expression (Ishibazawa et al., 2011) and NO bioavailability (Rodríguez and González, 2014). Exercise programs are one of the best-suited approaches to prevent CV comorbidities and a subsequent stroke (Tang et al., 2009; Jurczak et al., 2014; Kirk et al., 2014; Marzolini et al., 2014; Prior et al., 2017), however, different exercise modalities and intensities, such as endurance *versus* resistance and low *versus* high intensities, could elicit different CV outcomes. Moreover, and to the best of our knowledge, there are no studies regarding carotid ESS during different modalities of exercises and intensities.

The purpose of this study was to determine exercise-induced blood flow patterns across different exercise modalities at three different intensities in the carotid artery. It was hypothesized that ESS and turbulent flow in the carotid artery would increase in an intensity-dependent manner and that exercises involving larger and more muscle groups would have larger ESS and more turbulent flow.

## Methods

### Experimental Design

Twenty participants were recruited for a repeated-measures study design. Participation within the study involved 2 sessions for maximal testing and 2 sessions of submaximal testing with 24–48 h between sessions. A priori power analysis was conducted in Rstudio using R statistical programing language and the “pwr” library; a total of 14 subjects with stratification by sex (7 per group) at an alpha level (α) of 0.05 with a large effect size (f) of 0.4, was determined to be enough to obtain power (β) of 0.80. All study protocols were in accordance with the Declaration of Helsinki and were approved by the Institution Review Board at the University of Texas at El Paso (Reference number: 1250657). All participants signed an informed consent form before engaging in their first testing session. Females were tested within an 8-day period, spanned from 4 days before to 4 days after the start of menses, to reduce any hormonal influence on vascular response (Adkisson et al., 2010; Mattu et al., 2020).

### Study Protocol

**FIGURE 1:**
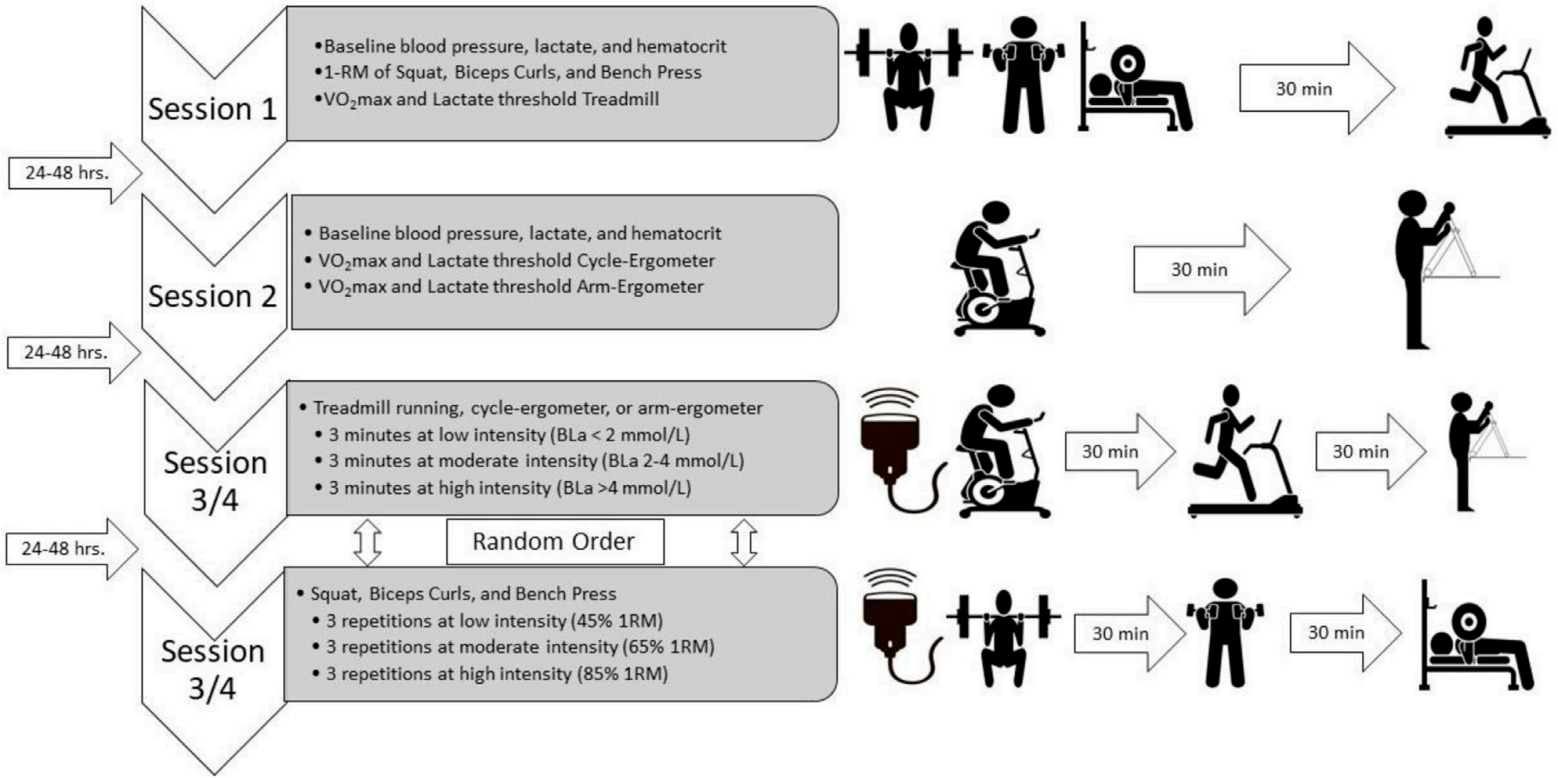
Study design. Four exercise sessions, 2 for maximal tests and 2 for submaximal tests, with 24–48 h between sessions. Ultrasound assessment during both submaximal sessions and exercise modalities sets were randomly assigned to either session 3 or session 4. VO2max, maximal oxygen consumption; BLa, blood lactate levels.

All testing was performed in a temperature-controlled room (24°C–26°C) and participants were asked to refrain of food, alcohol, and smoking for at least 8 h before any testing session. Participants completed demographic and screening questionnaires to determine eligibility. Height and mass were taken using a calibrated stadiometer and scale, respectively (Detecto PHR, Detecto, Webb, MO, United States). Then, resting blood pressure was obtained using an automated brachial blood pressure cuff (BP760, Omnron Healthcare, Inc., Lake Forest, IL, United States). In addition, and at the beginning of every visit, hematocrit (HemataStat II Hematocrit Analyzer, Separation Technology Inc., Sanford, FL, United States) and resting blood lactate (BLa) levels (Lactate Plus, Nova Inc., Boston, MA, United States) were obtained from the lower end of the earlobe as previously described (Rascon et al., 2020; Gurovich et al., 2021a). Then, for session 1, subjects completed 3 maximal strength tests (Squat, Bench Press, and Biceps curls), then subjects rested for at least 30 min (Tagesson and Kvist, 2007; Neto et al., 2015; García-Ramos et al., 2019) and performed a graded exercise test on the treadmill (Trackmaster TMX58, Newton, KS, United States) to determine maximal oxygen consumption (VO_2_max) and lactate threshold. In addition, and to confirm recovery, BLa levels were obtained after the 30 min resting period and participants were not allowed to perform the next exercise testing if BLa leveles were not back to baseline levels. In session 2, participants performed two other graded exercise tests in the cycle-ergometer (Corival, Lode, Groningen, Netherlands) and arm-ergometer (Angio, Lode, Groningen, Netherlands) with at least 30 min between tests. All three VO_2_max tests included blood draws from the earlobe to determine BLa at the end of each exercise stage. The 6 sub-maximal exercises (i.e., Squat, Bench Press, Biceps curls, treadmill, cycling, and arm-ergometer) were randomly assigned to sessions 3 and 4, and performed each exercise at three different exercise intensities. Participants performed three repetitions of Squat, Bench Press, and Biceps curls at low (45% 1-RM), moderate (65% 1-RM), and high intensity (85% 1-RM) each and 3-min steady-state exercise stages of treadmill, cycling, and arm-ergometer at low (BLa < 2 mmol/L), moderate (BLa 2–4 mmol/L), and high intensity (BLa > 4 mmol/L) (Rascon et al., 2020) (Figure 1). At least 30 min between sub-maximal exercise sets were provided to recovery.

All three graded exercise tests used a protocol with speed/workload increased every 2-min (Beltz et al., 2016). VO_2_max was obtained using a metabolic cart (TrueOne 2400, Parvomedics Inc., Sandy, UT, United States). At 30 s before the end of each stage, BLa was drawn from the participant’s earlobe, to determine BLa threshold, along with reported heart rate and rate of perceived exertion. A successful trial was considered if the following criteria were met: 1) BLa > 8.0 mmol/L, respiratory exchange ratio (RER) > 1.10, heart rate was within 10 bmp of estimated maximal heart rate (220—age), and RPE > 17 (Beltz et al., 2016).

The 1-RM testing consisted of a familiarization and technique inspection of the individual’s exercise execution. Thereafter, participants were asked to predict the maximal load they could achieve. Then, participants performed 5–10 repetitions of the predicted load at a comfortable pace. The load was increased by 20% for the following set and performed for 2–3 repetitions. Then load was increased by 2.5–5 kg until participants reached failure (Seo et al., 2012; Montalvo et al., 2021). Technical execution analysis, as well as spotting, was performed by a Certified Strength and Conditioning Specialist (SM).

### Blood Flow Pattern Testing

**FIGURE 2:**
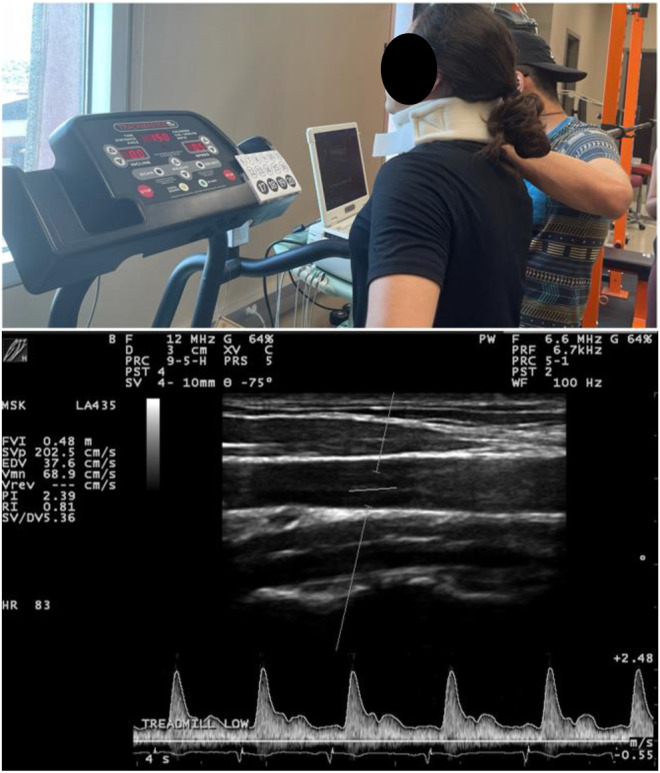
Typical ultrasound testing setup with neck probe holder utilized during all exercise testing and representative ultrasound image.

During sub-maximal exercise sets (sessions 3 and 4), real-time carotid artery longitudinal images and blood flow velocity were recorded with a 12 MHz ultrasound transducer and Doppler, (LA435, MyLab30 Gold, Esaote, Firenze, Italy), which has secure with a cervical probe holder placed on the participant’s neck as previously described (Gurovich et al., 2021b; Morales-Acuna et al., 2020). Ultrasound images and Doppler signals were obtained on the common carotid artery 2 cm below the bifurcation of the anterior and posterior carotid arteries and then analyzed with edge detection technology (Vascular Analysis Integrative System, Medical Imaging Applications, Coralville, IA, United States) and a data acquisition system (MP150WSW, BIOPAC Systems Inc., Goleta, CA, United States) (Figure 2). ESS was obtained by Womersley’s approximation and the presence of turbulent flow *via* Reynold’s number (Re) as previously described (Gurovich and Braith, 2012; Morales-Acuna et al., 2019; Rascon et al., 2020; Gurovich et al., 2021a). The presence of laminar or turbulent flow was defined *via* Re, where undisturbed laminar flow values were < 200, disturbed blood flow values between 200–1800, and turbulent flow values > 2000 (Davies, 2009). Both ESS and Re were determined within a single cardiac cycle to minimize the effects that heart rate and cardiac output have in ESS, as previousle described (Gurovich and Braith, 2012; Morales-Acuna et al., 2019; Rascon et al., 2020; Gurovich et al., 2021b).

### Statistical Analysis

Data were compiled into a master data spreadsheet (Excel, Microsoft 2021). Data were then exported into Rstudio Integrative Development Environment (Rstudio, 2020) and analyzed using a custom-built script in R statistical programming language (R 4.1.2). The “dplyr” package was used for grammar data manipulation, “forecats” for factor re-leveling, “ggplot2” and “ggpurb” for data visualization, “psych” for data descriptives, “cvcqv” for reliability analysis, “lm4” and “lmerTest” for linear mixed-effects models, “rstatix” for post-hoc pairwise comparisons, and effect sizes. Data distribution was assessed *via* Shapiro-Wilk test. Baseline demographic data were analyzed by a series of independent t-tests between males and females. Reliability of baseline ESS and Re were analyzed using a coefficient of variation (CV) and interpreted as < 10% as very good, 10%–20% as good, 20%–30% as acceptable, and >30% as poor (Campbell et al., 2010). Differences between exercise modalities and intensities were assessed using a general linear mixed-effects model for repeated measures with adjusting for individual differences as a random effect; the model was as follows: dependent variable ∼ exercise modality + exercise intensity + Sex + exercise modality*exercise intensity + (1|Participant). Pairwise differences were analyzed post-hoc with a Holm-Bonferroni *p*-value correction (*p.adj*) when appropriate; the effect size was obtained through standardized mean differences using Cohen’s D with a Hedge’s g (ES_g_) correction for a small sample size, and interpreted as follows: ES_g_ < 0.2 as very small, 0.2–0.49 as small, 0.5–0.79 as moderate, and > 0.8 as large (Hopkins, 2009). Statistical significance was set *priori* at an alpha level of 0.05. Re was analyzed by visual analysis using a 95% confidence interval (CI) as previously described (Gurovich et al., 2021a). Data and data analysis scripts are available in a repository for data analysis replication in https://github.com/Samuelmontalvo/Modalities.

## Results

Out of the 20 participants, 6 were unable to finish all 4 visits due to the COVID-19 lockdown. Hence, only 14 participants were able to complete the study. All data analyzed was normally distributed. Demographics and descriptive data for the final participants are provided in Table 1. Males were taller, had a higher VO_2_max on treadmill (*t* = 2, *p* = 0.002), 1-RM Bench Press (*t* = 6, *p* < 0.01), and 1-RM Biceps curls (*t* = 3, *p* = 0.02) than females. Baseline reliability analysis showed a good inter-testing reliability on ESS [CV = 16.9 (95%CI = 12.3–21.5)] and acceptable inter-testing reliability on Re [CV = 22.7 (95%CI = 16.4–29.1)].

**TABLE 1:** Demographic and descriptive data of the participants.

	All Mean ± SD	Males Mean ± SD	Females Mean ± SD	t	p
Age (yrs.)	23.00 ± 2.86	24.00 ± 3.56	22.00 ± 1.63	1.35	0.21
Height (m)	1.66 ± 0.09	1.73 ± 0.05	1.60 ± 0.08	3.48	<0.01
Weight (kg)	69.18 ± 11.03	73.94 ± 7.60	64.41 ± 12.37	1.73	0.11
BMI (kg/m2)	24.97 ± 3.45	24.73 ± 2.19	25.21 ± 4.57	0.25	0.80
SBP	113.29 ± 8.91	117.00 ± 9.07	109.57 ± 7.59	1.66	0.12
DBP	74.07 ± 6.83	75.14 ± 7.49	73.00 ± 6.51	0.57	0.57
Treadmill VO2 (ml/kg/min)	43.26 ± 9.99	50.6 ± 5.21	35.91 ± 7.95	4.08	<0.01
Cycle-ergometer VO2 (ml/kg/min)	32.00 ± 9.18	34.59 ± 10.16	29.41 ± 7.99	1.05	0.31
Arm-ergometer VO2 (ml/kg/min)	28.74 ± 9.47	32.34 ± 10.43	25.13 ± 7.43	1.49	0.16
1RM-Squat (kg)	83.34 ± 36.84	101.83 ± 43.04	64.86 ± 17.08	2.11	0.06
1RM-Bench (kg)	55.78 ± 24.97	76.86 ± 16.18	34.70 ± 7.26	6.28	<0.01
1RM-Biceps (kg)	33.73 ± 20.28	47.02 ± 21.36	20.43 ± 4.73	3.21	0.01

Overall, the model indicated a main effect of exercise modality (F_(2,247)_ = 53.78, *p* < 0.001) and intensity (F_(2,247)_ = 63.16, *p* < 0.001), and a significant intensity * modality interaction (F_(10,247)_ = 2.99, *p* < 0.01) on ESS (Table 2). However, there was no main effect of sex on ESS (F_(1,12)_ = 2.01, *p* = 0.18) or Re (F_(1,12)_ = 0.12, *p* = 0.73). Moreover, there was a significant random effect (*p* < 0.001), indicating significant individual variability in ESS. Due to the no effect of sex within our model, post-hoc pairwise comparisons were performed with all individuals as one group.

**TABLE 2:** Endothelial Shear Stress (in dynes/cm^2^) by exercise intensity and modality.

Rest		Low intensity		Moderate intensity		High intensity		Effect or interaction	
Modality	Mean ± SD	Modality	Mean ± SD	Modality	Mean ± SD	Modality	Mean ± SD	F	*p*
Baseline	23.8 ± 4.8	Arm-ergometer	39.4 ± 10.7	Arm-ergometer	47.8 ± 12.1	Arm-ergometer	57.8 ± 20.7	Intensity	
Baseline two	26.5 ± 3.3	Cycle-ergometer	48.0 ± 10.8	Cycle-ergometer	62.6 ± 19.4	Cycle-ergometer	77.5 ± 20.3	63.16	<0.01
—	—	Treadmill	47.5 ± 13.1	Treadmill	67.3 ± 17.9	Treadmill	84.7 ± 9.7	Modality	
—	—	Bench press	34.8 ± 13.0	Bench press	42.1 ± 12.0	Bench press	45.6 ± 13.7	53.79	<0.01
—	—	Biceps curls	37.3 ± 13.3	Biceps curls	41.3 ± 12.8	Biceps curls	50.7 ± 14.7	Intensity*Modality	
—	—	Squat	44.1 ± 14.4	Squat	48.8 ± 16.4	Squat	56.8 ± 13.57	2.99	<0.01

**FIGURE 3:**
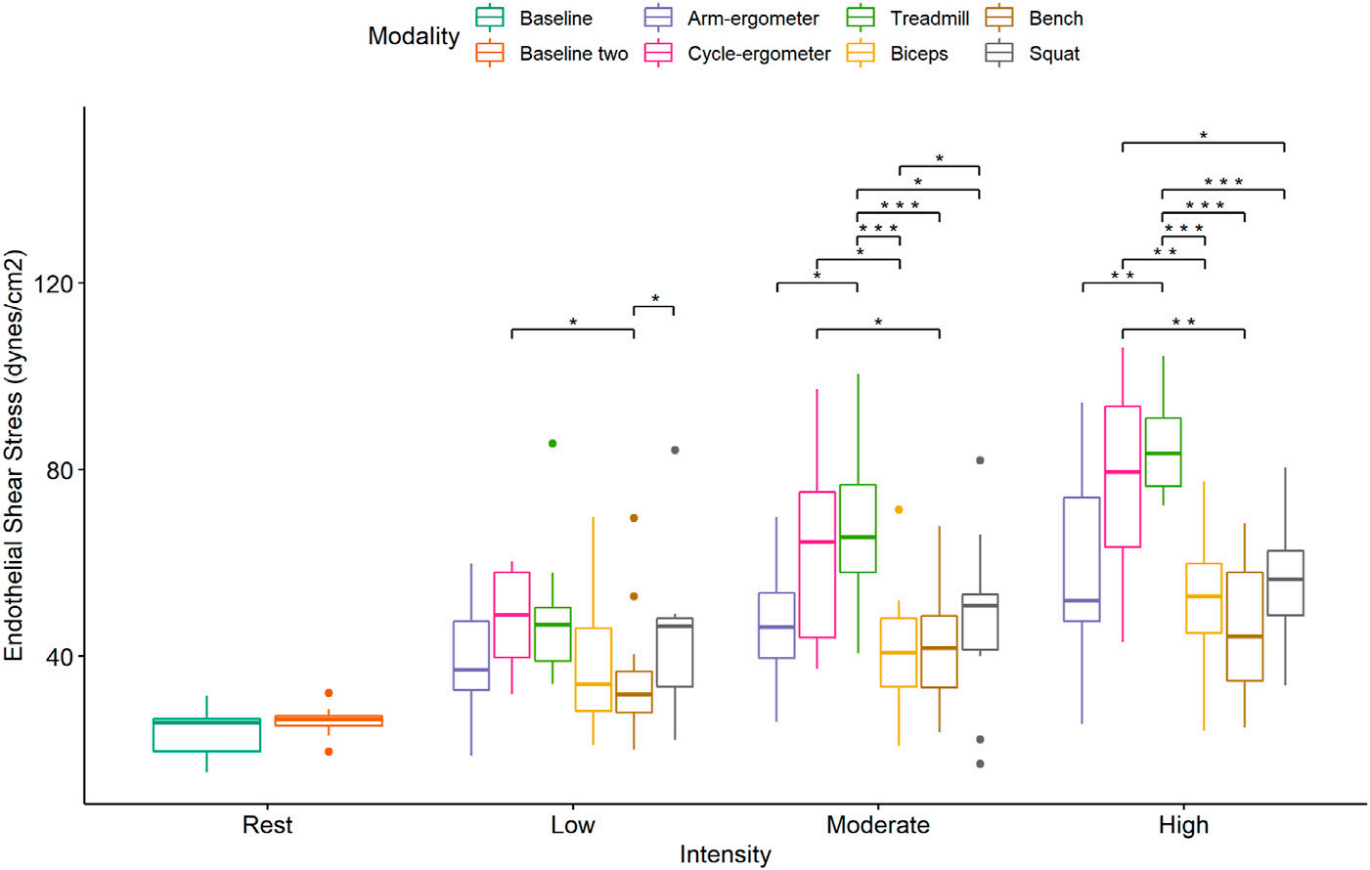
Boxplot of Endothelial Shear Stress (ESS) by exercise modality and intensity with comparisons between modalities at each intensity. *p < 0.05; **p < 0.01; ***,*p* < 0.001.

Post-hoc pairwise analysis within exercise modalities showed that almost all exercise modalities were influenced by intensity (*p* < 0.01) with large effect size between intensities (Figure 2; Table 3). Only Squat at low intensity vs. moderate intensity [*t* = -1.17, p.adj = 0.26, ES_g_ (*small*) = -0.29], Bench Press at low intensity vs. high intensity [*t* = -2.08, p.adj = 0.12, ES_g_(*moderate*) = -0.52], and Bench Press at moderate intensity vs. high intensity [t = -0.81, p.adj = 0.43, ES_g_(*small*) = -0.20] were not statistically different (Figure 3; Table 3).

**TABLE 3:** Pairwise comparisons between exercise modalities by exercise intensity for endothelial shear stress (ESS).

Intensity	Modality 1	Modality 2	t	p.adj	Hedges g	Effect
Low	Cycle-ergometer	Bench	3.639	0.042	0.915	Large
Low	Squat	Bench	3.950	0.025	0.994	Large
Moderate	Cycle-ergometer	Bench	3.739	0.022	0.940	Large
Moderate	Cycle-ergometer	Biceps	3.847	0.022	0.968	Large
Moderate	Treadmill	Arm-ergometer	4.081	0.017	−1.027	Large
Moderate	Treadmill	Squat	3.965	0.019	0.997	Large
Moderate	Treadmill	Bench	6.723	0.000	1.691	Large
Moderate	Treadmill	Biceps	5.742	0.001	1.444	Large
Moderate	Squat	Biceps	3.811	0.022	0.959	Large
High	Cycle-ergometer	Squat	3.382	0.044	0.851	Large
High	Cycle-ergometer	Bench	5.218	0.002	1.312	Large
High	Cycle-ergometer	Biceps	4.548	0.005	1.144	Large
High	Treadmill	Arm-ergometer	5.283	0.002	−1.329	Large
High	Treadmill	Squat	7.487	0.000	1.883	Large
High	Treadmill	Bench	7.289	0.000	1.834	Large
High	Treadmill	Biceps	7.725	0.000	1.943	Large

**FIGURE 4:**
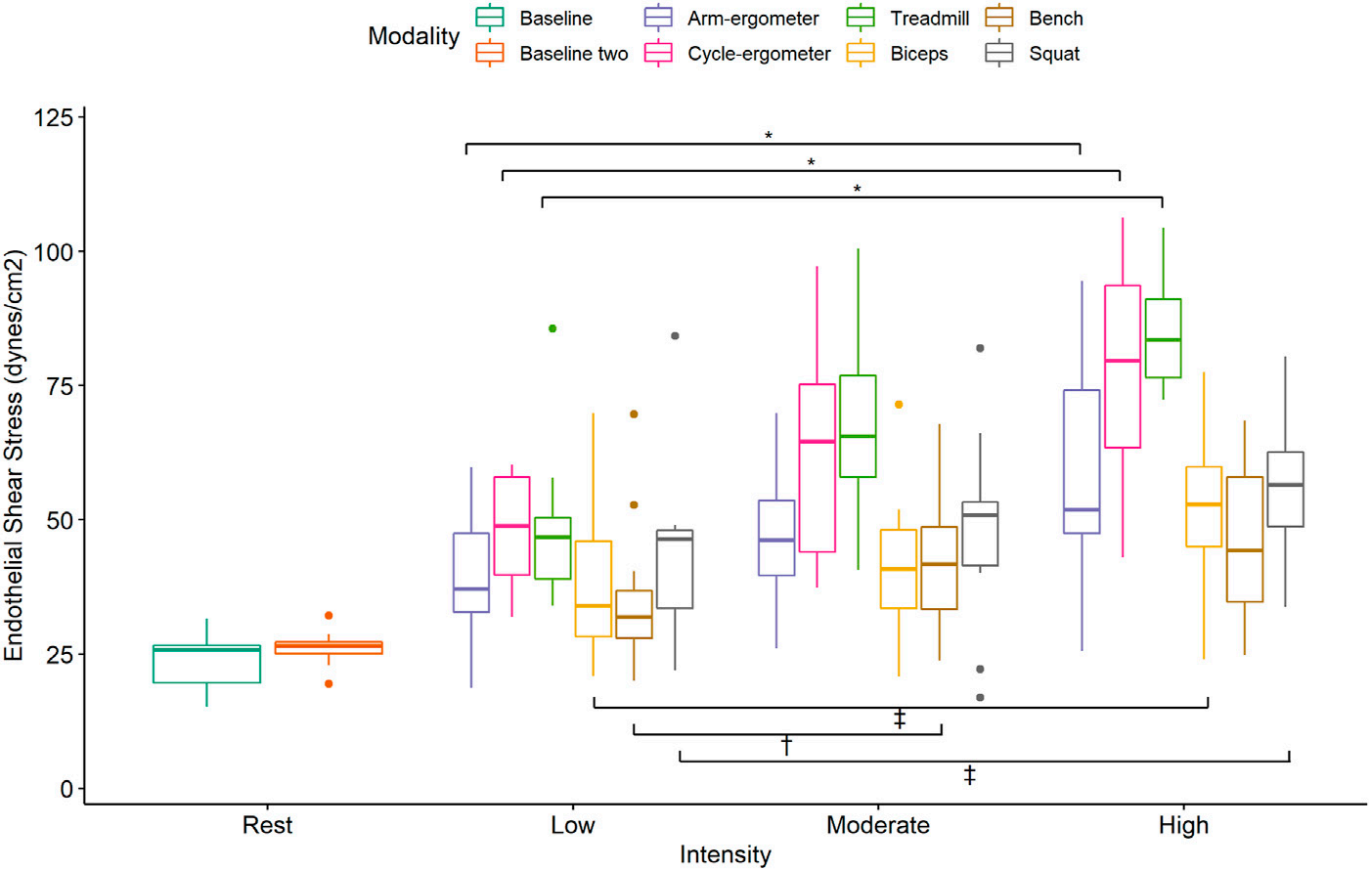
Boxplot of Endothelial Shear Stress (ESS) by exercise modality and intensity with comparisons within modalities at each intensity. *, *p* < 0.05 low vs. moderate, low vs. high, and moderate vs. high; †, *p* < 0.05 low vs. moderate Bench press; ‡, *p* < 0.05 low vs. high and moderate vs. high; †, *p* < 0.05 low vs. moderate Biceps and Squat.

Pairwise comparisons for ESS during low-intensity exercise showed significant differences and large effects between cycle-ergometer vs. Bench Press and Squat vs. Bench Press (Figure 4; Table 4). Similarly, there were significant differences and large effects at moderate exercise intensity between treadmill vs. arm-ergometer, treadmill vs. Squat, treadmill vs. Bench Press, treadmill vs. Biceps curls, and Squat vs. Biceps curls (Figure 4; Table 4). Finally, there were significant differences and large effects at high exercise intensity between cycle-ergometer vs. Squat, cycle-ergometer vs. Bench Press, cycle-ergometer vs. Biceps curls, treadmill vs. arm-ergometer, treadmill vs. Squat, treadmill vs. Bench Press, and treadmill vs. Biceps curls.

**TABLE 4:** Pairwise comparisons within exercise modality by intensity for endothelial shear stress (ESS).

Modality	Intensity 1	Intensity 2	t	p.adj	Hedges g	Effect
Cycle-ergometer	Low	Moderate	−4.93	0.00	−1.24	large
Cycle-ergometer	Low	High	−8.61	0.00	−2.16	large
Cycle-ergometer	Moderate	High	−7.14	0.00	−1.80	large
Arm-ergometer	Low	Moderate	−3.68	0.01	−0.93	large
Arm-ergometer	Low	High	−5.07	0.00	−1.28	large
Arm-ergometer	Moderate	High	−3.11	0.01	−0.78	moderate
Treadmill	Low	Moderate	−4.58	0.00	−1.15	large
Treadmill	Low	High	−8.18	0.00	−2.06	large
Treadmill	Moderate	High	−3.60	0.00	−0.91	large
Squat	Low	Moderate	−1.17	0.26	−0.29	small
Squat	Low	High	−3.79	0.00	−0.95	large
Squat	Moderate	High	−4.02	0.00	−1.01	large
Bench	Low	Moderate	−4.67	0.00	−1.18	large
Bench	Low	High	−2.08	0.12	−0.52	moderate
Bench	Moderate	High	−0.81	0.43	−0.20	small
Biceps	Low	Moderate	−2.19	0.05	−0.55	moderate
Biceps	Low	High	−5.81	0.00	−1.46	large
Biceps	Moderate	High	−5.24	0.00	−1.32	large

**FIGURE 5:**
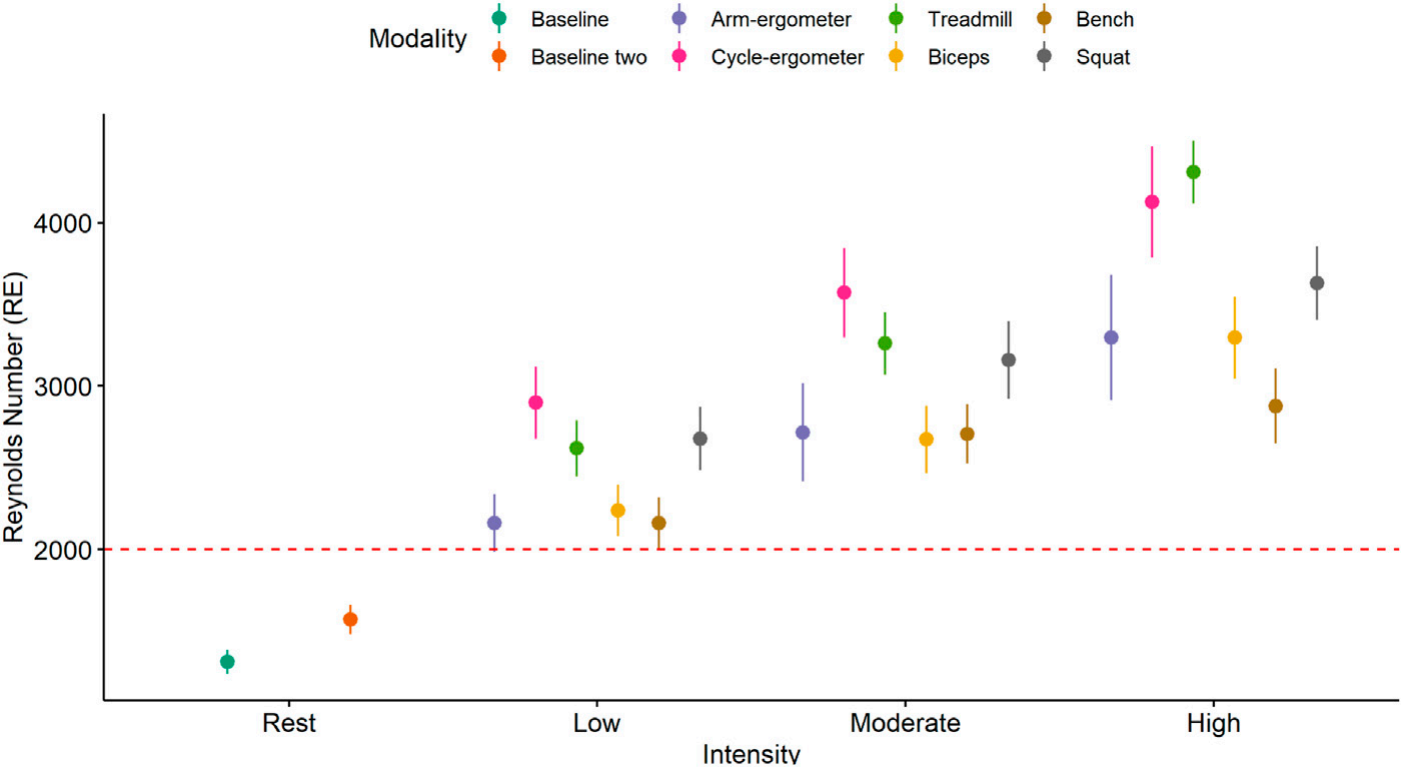
Mean and 95% CI of Reynolds number (Re) by exercise modality and intensity with a turbulent flow threshold number of 2,000 (red dash line).

Visual analysis of the Re plot using mean and error plot indicates that all exercise modalities from low to high intensity resulted in turbulent flow (Re > 2000) (Figure 5).

## Discussion

The purpose of the current study was to determine the effects of different exercise modalities at three different exercise intensities on endothelial shear stress (ESS) and blood flow patterns (presence of turbulent flow) at the carotid artery. Our primary hypothesis was confirmed as exercises involving larger and more muscle groups, like running on a treadmill, at higher intensities produced greater ESS and turbulent blood flow than other exercise modalities with less muscle recruitment, like Biceps curls, and lower intensities. However, exercise duration could be a confounding factor. In addition, the results of the present study confirmed that ESS increases in an intensity-dependent manner regardless of the exercise modality and that blood flow is mainly turbulent regardless of exercise modality or exercise intensity.

Approximately 40%–60% of the beneficial effects of exercise training in preventing/treating cardiovascular disease, including stroke, are unrelated to the reduction in traditional cardiovascular risk factors (Mora et al., 2007; Green, 2009). Several authors (Laughlin, 1995; Hambrecht et al., 2000; Green et al., 2002; Green et al., 2008; Laughlin et al., 2008; Green, 2009) have established a close relationship between exercise training and improvements in endothelial function. In addition, previous studies have shown that the lack of or low ESS can result in vascular inflammation, upregulation of matrix-degrading proteases, and arterial wall remodeling, which promotes the transition of stable to unstable plaque in atherosclerotic lesions (Koskinas et al., 2009). Moreover, it has been reported that low ESS and oscillatory shear stress can result in atherosclerotic lesions due to plaque formation (Cheng et al., 2006). Therefore, it is possible to speculate that the direct mechanical effects of exercise-induced blood flow patterns on the vascular endothelium could be a major mitigating factor in the prevention of cardiovascular disease.

The results of the current study showed that exercise-induced blood flow patterns are associated with exercise modality. For example, running on a treadmill or cycling at high intensity elicits more ESS than any of the resistance exercises at a comparable high intensity (Figure 3). This difference can be attributed to a larger muscle mass recruitment during running or cycling when compared to a single resistance exercise. In addition, ESS is intensity-dependent as almost all exercise modalities showed an increase in ESS when intensity is increased (Figure 4). This finding might be also associated with an increase in muscle recruitment as higher intensities would recruit a larger percentage of muscle mass. Moreover, and interestingly, it appears that all exercise modalities produced turbulent flow in the carotid artery, regardless of the exercise intensity (Figure 5). The presence of turbulent flow during exercise could be explained by the rather larger size of the carotid artery, compared to the brachial artery (Gurovich and Braith, 2012), and the systematic increase in blood flow velocity with the increased exercise intensity (Nichols and O'Rourke, 2005). Finally, the results of the current study showed no sex differences in any of the exercise modalities at any of the intensities. This is consistent with previous findings from our laboratory when assessing the brachial artery during cycle-ergometry at low, moderate, and high intensities (Gurovich et al., 2021b).

Even though the current study is not the first one assessing carotid blood flow during exercise (Babcock et al., 2015; Hellstrom et al., 1996; Jiang et al., 1995; Liu et al., 2015; Sato et al., 2011; Sato and Sadamoto, 2010; Wang et al., 2019), this is the first study comparing different exercise modalities and intensities. Previous studies have predominantly used walking/running on a treadmill or cycling, both upright and recumbent. For example, Jiang et al. (Jiang et al., 1995) assessed carotid blood flow velocity in eight healthy male participants during a graded exercise test on a treadmill. Unfortunately, the authors were not able to determine ESS as they found some technical difficulties with ultrasound imaging during their protocol. These technical difficulties were avoided in the current study by using a customized, patent-pending cervical probe holder placed on the participant’s neck (Gurovich et al., 2021a; Morales-Acuna et al., 2020). Nevertheless, Jiang et al. found a significant increase in carotid artery blood flow velocity, up to 52% from baseline values, during exercise. Similar results were found during cycling at submaximal intensities in healthy men (Hellstrom et al., 1996), healthy women (Sato and Sadamoto, 2010), and healthy men and women (Sato et al., 2011). Similar to the findings of the present study, all these studies showed an intensity-depend increase in carotid artery blood flow, ranging from 17% to 42%, with submaximal exercise. Interestingly, Babcock, Heffernan, et al. (Babcock et al., 2015) measured carotid blood flow before and after a short bout (e.g., 30 s) of maximal exercise in 55 healthy adults, with different exercise backgrounds. Their findings showed a 15%, 19%, and 19% increase in mean carotid blood flow velocity, mean carotid blood flow, and mean shear rate, respectively. These rather smaller increases after maximal exercise could be explained by the short bout of exercise as 30 s might not be enough to elicit other vascular acute adaptations. Even though shear rate might reflect ESS, the results of the current study showed an increase in ESS from 39%, during Bench Press at low intensity, to 239%, during treadmill running at a high intensity (Table 2). These findings may confirm that longer exercise bouts could elicit different acute vascular responses and that shear rate should not be considered as a surrogate for ESS (Gurovich and Braith, 2012). The ESS data shown in the current study are in agreement with previous reports (Liu et al., 2015; Wang et al., 2019). Both Wang et al. (2019) and Liu et al. (2015) reported an increased ESS, from 40% to 100%, during exercise. In addition, Wang et al. (2019) showed an intensity-dependent increase in ESS from resting to moderate and high intensity cycling exercise (∼50 dynes/cm^2^ vs. ∼75 dynes/cm^2^ vs. 100 dynes/cm^2^, respectively) that are comparable to the cycling ESS in the current study (resting: 26.5 ± 3.3 dynes/cm^2^, low: 48.0 ± 10.8 dynes/cm^2^, moderate: 62.6 ± 19.4 dynes/cm^2^, and high: 77.5 ± 20.3 dynes/cm^2^). Interestingly, the resting data from the current study is very similar to data obtained with echo particles imaging velocimetry (PIV) (Gates et al., 2018); however, exercise PIV data has yet to be determined.

Even though the current study was designed to assess blood flow patterns during an acute bout of exercise, these findings can be associated with adaptations such as endothelial function and atherosclerotic plaque vulnerability. For example, there are some conflicting results when comparing endothelial function before and after resistance and aerobic exercises (Iwamoto et al., 2018; Boeno et al., 2019). Boeno et al. (2019) showed no improvement in endothelial function, measured *via* flow-mediated dilation, with a single session of repeated knee extension exercise at a moderate and high intensity. In contrast, Iwamoto et al. (2018) showed that endothelial function improved in an intensity-dependent manner after low intensity (50%–55% of HRmax) and high intensity (75%–80% HRmax) cycle-ergometry. Similarly, Spence et al. showed that 6 months of aerobic and resistance training induced significant changes in the carotid artery size and function (Spence et al., 2013). In addition, there is some evidence that turbulent flow can improve the strength of atherosclerotic plaque (Cheng et al., 2006; Koskinas et al., 2009). Both Cheng et al. (2006) and Koskinas et al. (2009) using very elegant study designs, showed that turbulent flow in pro-atherosclerotic vascular areas can induce stable lesions by mobilizing smooth muscle cells. Altogether, the significant increase in ESS and turbulent flow at higher intensities observed in the current study, if applied chronically (i.e., exercise training), may elicit beneficial effects to treat and prevent cardiovascular diseases.

### Limitations

The present study is not exempt from limitations. Our study was limited to the sample size. Our between-subjects comparison analyzes could have been compromised by the low sample size (males = 7, females = 7). However, because of inexistence differences between males and females in blood flow patterns, our overall sample size was 14 participants, which was enough to show differences in responses through the standardized mean difference as denoted by the effect size. Moreover, each of the pairwise comparisons (exercise modality by intensity) yielded a possible 42 comparisons. Thus, in order to avoid the increased chance of committing type 1 (false positive) and type 2 (false negative) errors, we utilized a Holm-Bonferroni correction (Eichstaedt et al., 2013). Another possible limitation is the difference in the exercise duration of each exercise modality. Future studies should use standardize time/volume of each exercise bout to make it more comparable.

The inferences derived from this investigation can only be extrapolated to a similar population (healthy young male and female participants), and the effects of different exercise modalities and intensities on ESS and Re for clinical populations (i.e., CV problems) remain unknown. Moreover, it is unknown if other alternative exercise modalities such as plyometrics (jumping), boxing, agility training, balance, Taichi, Yoga, etc,. would affect (short or long term) endothelial shear stress and function, and as such, researchers should investigate these. Finally, our study was cross-sectional, and only acute interaction of exercise modality and intensity and ESS or Re can be inferred. Thus, the differences between exercise modalities and intensities on ESS or Re at short and long-term exercise remains unknown.

## Conclusion

Blood flow patterns during exercise in the carotid artery show that flow is mainly turbulent, independent of the exercise modality and intensity and that ESS is dependent on exercise intensity regardless of the exercise modality. In addition, activities engaging larger and more muscle groups, like running or biking, at a high intensity yield the greatest ESS. Thus, clinicians should take into consideration exercise-induced blood flow patterns at the carotid artery during the different exercise intensities and modalities.

## Data Availability

The datasets presented in this study can be found in online repositories. The names of the repository/repositories and accession number(s) can be found below: https://github.com/Samuelmontalvo/Modalities.
